# Functional improvement in children and adolescents with primary headache after an interdisciplinary multimodal therapy program: the DreKiP study

**DOI:** 10.1186/s10194-022-01481-1

**Published:** 2022-08-25

**Authors:** Hanna Sobe, Matthias Richter, Reinhard Berner, Maja von der Hagen, Antje Hähner, Ingo Röder, Thea Koch, Rainer Sabatowski, Anna Klimova, Gudrun Gossrau

**Affiliations:** 1 Interdisciplinary Pain Center, University Hospital and Faculty of Medicine Carl Gustav Carus, TU Dresden, Fetscherstr. 74, 01307 Dresden, Germany; 2 Department of Pediatrics, University Hospital and Medical Faculty Carl Gustav Carus, TU Dresden, Dresden, Germany; 3 Abteilung Neuropädiatrie, Medizinische Fakultät Carl Gustav Carus, TU Dresden, Dresden, Germany; 4 Smell & Taste Clinic, Department of Otorhinolaryngology, TU Dresden, Dresden, Germany; 5 NCT Partner Site Dresden, Institute for Medical Informatics and Biometrics, Faculty of Medicine Carl Gustav Carus, TU Dresden, Dresden, Germany; 6 Department of Anesthesiology and Intensive Care, University Hospital and Faculty of Medicine Carl Gustav Carus, TU Dresden, Dresden, Germany

**Keywords:** Headache, Children, Therapy program

## Abstract

**Background:**

More than 2/3 of children and adolescents in Germany regularly suffer from headaches. Headache-related limitations in everyday life, school drop-out and educational impairment are common. Structured therapy programs for young headache patients are widely missing.

**Methods:**

One hundred eleven patients with frequent migraine and/or tension type headache were treated in a 15 hour group program in afternoons, parallel with school, parents received 7 hours of therapy. At the beginning of the program (T0), 6 (T1) and 12 months (T2) after completion, data on headache related disability (PedMidas), headache frequency, intensity, and pediatric pain disability score (PPDI) were prospectively collected to investigate the effects of the therapy.

**Results:**

Seventy-five patients (9-19 years, median = 14; 66.7% female) and their parents provided patient reported outcome measures showing at T1 (65 patients) and T2 (47 patients) reduced headache frequency (last 3 months headache days median T0: 30 days; T1: 18 days, reduction of median 12 days since T0; T2: 13 days, reduction of median 17 days since T0). Linear mixed models revealed significant reduction (T0/T1 *p* = 0,002; T0/T2 p = 0,001). Reduced headache disability has been reported at T1 and T2 (PedMidas median T0 = 30, T1 = 15, T2 = 7; *p* < 0,001, p < 0,001 respectively). Follow up data of a subgroup of patients 24 months after the treatment point to sustainable effects.

**Conclusions:**

The interdisciplinary multimodal headache therapy program DreKiP reduces headache frequency and headache related disability significantly 6-12 months following its completion.

**Trial registration:**

DRKS00027523, retrospectively registered.

**Supplementary Information:**

The online version contains supplementary material available at 10.1186/s10194-022-01481-1.

## Background

Evidence-based treatment options in pediatric headache remain limited. Since headache is the most common reason for medical consultations and the most common pain disorder in children and adolescents, there is the need to improve the situation [1]. More than 2/3 of young people in Germany regularly suffer from headaches [2, 3]. The majority of adolescent migraine patients continue to suffer from migraine in adulthood [4–6]. Headaches in children are often not perceived as a serious disease and diagnosis and therapy are not consistently pursued. Investigations in community samples depicted female gender, greater socioeconomic impairment, depressive symptoms as negative prognostic factors in patients with frequent headaches in adolescents [7].

Young headache patients show high co-morbidity with further chronic pain [1, 8, 9]. Lifestyle factors as consumption of caffeine, lack of physical activity and psychosocial risk factors (e.g. family conflicts) are associated with frequent headaches in young patients [2, 5, 8, 10].

Approximately 5% of children and adolescents with headaches show severe headache-related limitations in everyday life from school drop-out to severe educational impairment [5, 11–13]. Especially for children and adolescents with headache-related restrictions of daily activities, a therapy that adresses the biopsychosocial dimension of chronic headaches is essential. With changes in behaviour, relaxation ability, physical activity due to recurrent headaches, interdisciplinary therapy strategies are necessary, including education, stress management, relaxation techniques and physical activation. Unimodal therapy strategies are usually not successful in this complex situation [6, 14, 15] .

Therefore, we set out to develop an interdisciplinary multimodal outpatient therapy program, consisting of headache education and a variety of practical therapies, enabling individual approaches for young headache patients [16]. Primary aim of the study is reporting therapy-related change in headache frequency and headache-related disability 6 and 12 months after the therapeutic intervention.

## Methods

We performed a prospective, monocentric study in patients admitted to an interdisciplinary multimodal outpatient treatment program. The study protocol was approved by the Ethics Board of the Faculty of Medicine at the TU Dresden (protocol number EK-462122017). Detailed information about the data collected were given to all participants and parents and informed written consent was obtained. All aspects of the study were performed in accordance with the Declaration of Helsinki.

### Patients

One hundred eleven children and adolescents participated in the multimodal outpatient therapy program DreKiP (Dresden children/adolescents headache program) with age-matched groups from January 2018 to June 2021. Patients data were prospectively collected at the start of the program (T0), six (T1), twelve (T2) and twenty-four months (T3) after its completion. The analysis is based on the data of 75 patients, who provided patient reported outcome measures for at least one follow up visit. Therapy groups have been organized as a collaboration between Headache Outpatient Clinic, Pain Center and Department of Neuropediatrics, University of Dresden [16]. All patients have been assessed by an interdisciplinary team of pediatrician, neurologist and pediatric psychotherapist. Before participation in the program, patients underwent a detailed clinical examination and standardized psychological evaluation. Motivated patients with a confirmed diagnosis of a primary headache disorder according to the ICHD-III criteria [17] for at least 6 months at the age of 9 to 18 years, headache-related limitations in school attendance, daily activities, quality of life were admitted to the therapy program.

### Interdisciplinary multimodal outpatient therapy program DreKiP for children and adolescents

**Fig. 1:**
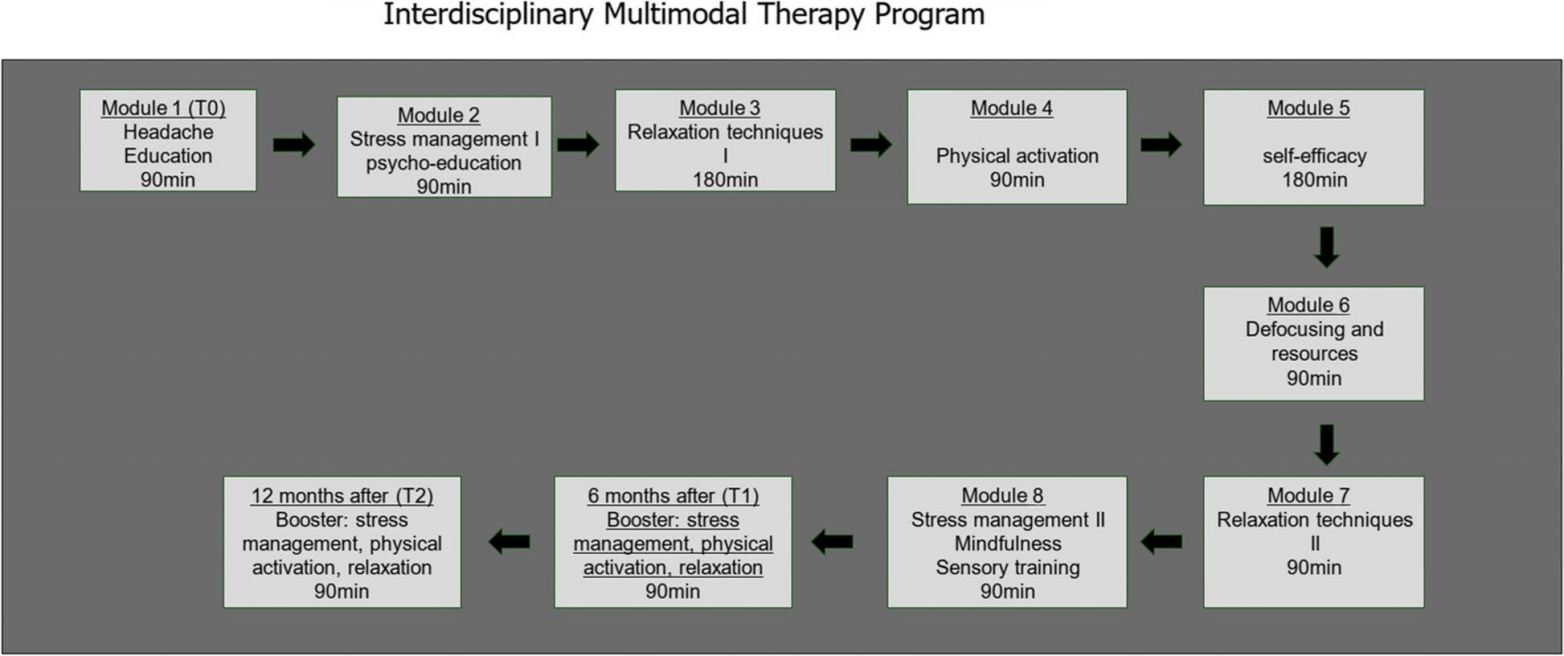
Overview of the interdisciplinary multimodal group therapy program

DreKiP consists of 8 therapy modules for patients including headache education, stress management, relaxation techniques, physical fitness, climbing therapy as communicative movement therapy to strengthen self-efficacy [18], art therapy, olfactory training as education and mindfulness training (Fig. 1) [16, 19]. Parent workshops focusing on education take place on four parallel dates.

DreKiP is carried out over 2-3 months in afternoons or weekends and interruption of school is not necessary. Each group consists of 4-8 patients of one age group. Patients receive a total of 15 hours of therapy and their parents 7 hours. The aim of the program is to educate the patients and their families about headaches and potential treatments, to recognize stressors and to practice stress management, relaxation techniques and defocusing exercises. Physical activation is encouraged as team sport and on the climbing wall. Self-efficacy is developed centrally and the everyday transfer of new knowledge is stimulated. For detailed contents of the therapy and booster modules 6 and 12 months after the program please see Supplementary Material 1.

### Clinical data and questionnaires

Clinical data as headache diagnosis, concomitant diseases and patient reported outcome measures (headache frequency and intensity, analgesic intake and questionnaires) have been prospectively collected. The pediatric migraine disability assessment score (PedMIDAS, range 0-240 points) [20] and pediatric pain disability score (PPDI) [21] have been used. PedMIDAS is a validated questionnaire to quantify functional impairment due to headache over a period of 3 months. Sum scores > 50 indicate severe, 31-50 moderate, 11-30 mild, 0-10 no or little impairment. PPDI is a short questionnaire for self- or parent assessment of pain-related impairments in children and adolescents with chronic pain.

### Statistical procedures and data analysis

The data analysis was performed using SPSS vs. 29 (SPSS Inc., Chicago, Illinois). Categorical variables are summarized as frequencies and percentages; continuous variables are reported as means (M) ± standard deviations (SD) or medians (with interquartile range). For continuous variables, the independent samples or paired t-test, Mann-Whitney U test, Wilcoxon sign-rank test, F-test (ANOVA), or Kruskal-Wallis test were employed, as appropriate. For categorical variables, the association was assessed using the chi-squared test, the differences in distributions between timepoints were investigated using the McNemar–Bowker test. For hypothesis testing, *p*-values less than 0.05 (two-sided) were considered statistically significant. An adjustment for multiple comparison was performed when necessary. For mixed-effects models, p-values were obtained using the Satterthwaite’s method.

## Results

### Patients in the program

**Fig. 2:**
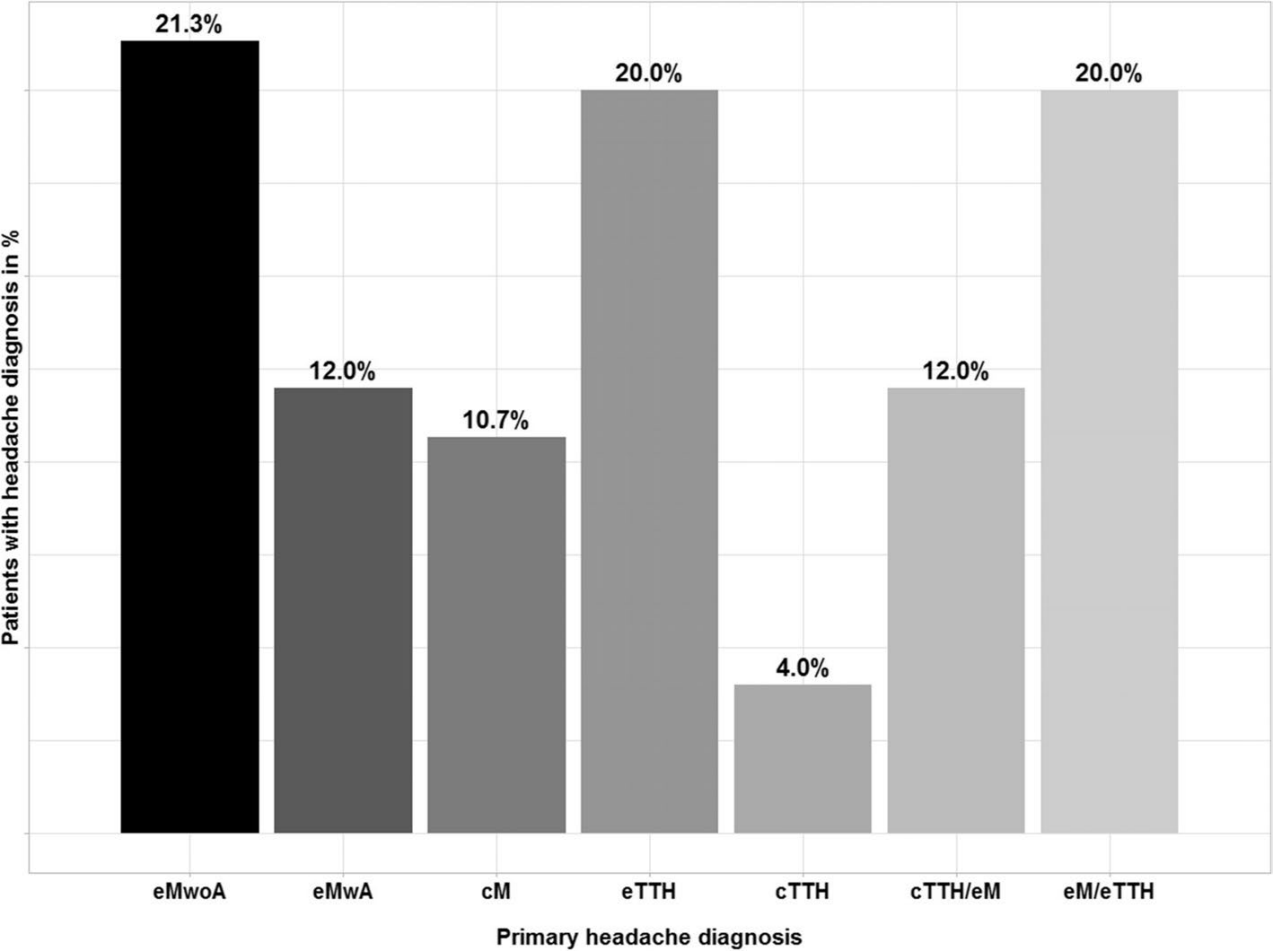
Primary headache diagnoses of patients in the program. 16 patients (21,3%) episodic Migraine without Aura (eMwoA), 9 patients (12%) episodic Migraine with Aura (eMwA), 8 patients (10,7%) chronic Migraine (cM), 15 patients (20%) episodic Tension Type Headache (eTTH), 3 patients (4%) chronic Tension Type Headache (cTTH), 9 patients (12%) chronic Tension Type Headache/episodic Migraine (cTTH/eM), 15 patients (20%) episodic Migraine/episodic Tension Type Headache (eM/eTTH)

Descriptive statistics and further results are reported for patients who provided outcome questionnaires for at least one follow up visit. This subcohort consists of 75 patients, 50/75 (67%) female, 9-19 years old (median = 14, IQR = 13:16; Supplementary Tables 1 and 2). All of the participants had at least one primary headache diagnosis according to the IHS criteria [22] (for details see Fig. 2).

In 45 (60%) participants, at least one accompanying chronic disease was diagnosed (Table 1).

**Table 1:** Frequency of additional diseases

	patients (n)	% of all patients
Patients with concomitant diseases	45	60
		% of patients with additional diseases
back or abdominal pain	18	40
Mental disorders	11	24
Organic brain disorders	9	20
Allergy/atopic disease	8	18
Endocrinological diseases	4	9
Ophthalmological disease	3	7
Other diseases	7	16

### Headache days at baseline and after group therapy

At the beginning of the program (T0), headache frequency data were available for 72 participants (96%). Patients reported having had on average 43.74 (±33.44) headache days within the last 3 months, with median 30 days (IQR = 15:90). Girls presented more headache days than boys: mean 48.4 (± 34.3) and 34.9 (±30.5), medians 40 (IQR = 15:90) and 20 (IQR = 15:40) days, respectively (W = 706, *p* = 0.158). Younger participants (≤13) showed on average 22 days less headache in the last 3 months than patients ≥14 years (W = 312, *p* = 0.008).

At T0, headache days differed significantly depending on the headache diagnosis (Kruskal-Wallis test, *χ*^2^ (3) = 16.194, *p* = 0.001).

Six months after the program (T1), 65 (86.7%) patients provided information about their headache days. The mean of 35.65(±34.19) days was reported (median = 18), with girls presenting on average 20.7 more headache days than boys (W = 635.5, *p* = 0.024). The overall difference between headache diagnoses was still considerable (Kruskal-Wallis test, *χ*^2^ (3) = 17.096, *p* < 0.001), but the difference between chronic and episodic migraineurs was not significant anymore (W = 51, *p*-value = 0.112). Chronic migraineurs improved and the headache frequency became lower.

**Fig. 3:**
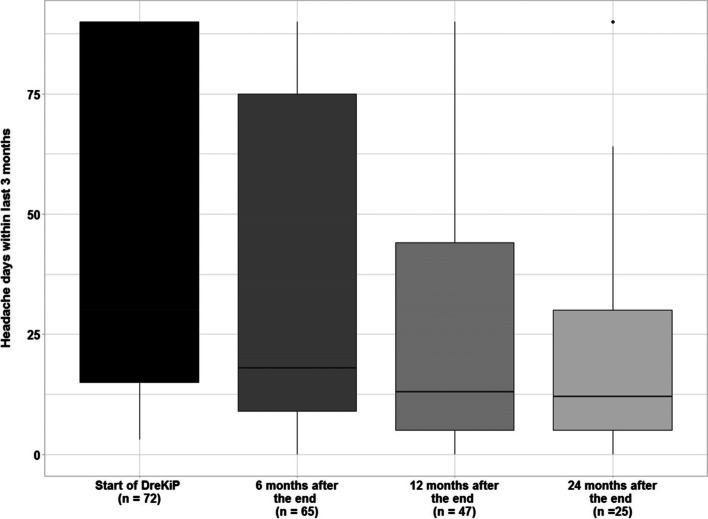
Headache frequency. (Median headache days in the last 3 months) at baseline and follow up 6, 12 and 24 months after the program “DreKiP” (stands for Dresden children and adolescent headache program)

Twelve months after completing the program (T2), data were provided by 47 (62.7%) patients. The mean number of headache days in the last 3 month was 27.7 (± 30.9), median 13, and thus overall reduced compared to baseline and to T1 (Fig. 3). No major differences between headache diagnoses were observed (Kruskal-Wallis, *χ*^2^ (3) = 6.595, *p* = 0.086).

Two-year follow-up data (T3) were provided by 25 (33.3%) patients. They had, on average, 23.32 (± 26.04) days, median = 12, which may indicate that the reduction achieved by 12 months remained stable. No significant differences between headache diagnoses were observed (Kruskal-Wallis, *χ*^2^ (3) = 0.145, *p* = 0.986).

Time trends in headache days were investigated using a linear mixed-effects model with random intercept per patient and fixed effects for gender, time point, and main diagnosis. This analysis arrived to similar conclusions, indicating a strong reduction in headache days. As compared to the baseline, the average number of headache days decreased on average by 11.2 days per last 3 months T1 (*p* = 0.002) and by 17.3 days per last 3 months at T2 (*p* < 0.001). Finally, at T3, patients experienced on average 16.7 days less per last 3 months compared to the baseline (*p* = 0.001). The interaction between time and gender was found borderline significant (*χ*^2^ (3) = 7.975, *p* = 0.047). In particular, the estimated number of headache days of girls after 6 months was about 16.8 days per last 3 months more than those of boys at the same time point (*p* = 0.029). However, no difference could be inferred after 1 year (*p* = 0.057) or 2 years (*p* = 0.526; Fig. 3).

### Headache intensity at baseline and after group therapy

At T0, 69 (92%) patients provided data describing their headache intensity. Seven of them (10.1%) reported having mild, 30 (43.5%) moderate, and 32 (46.4%) severe headache, and the intensity seems to be associated with headache diagnosis (*χ*^2^ (6) = 15.499, *p* = 0.017). While nobody reported having pain of a highest severity, patients with episodic migraine experienced severe headache more frequently than other patients (OR = 3.70, *p* = 0.026).

The intensity data of 57 (76%) patients were collected at T1. One adolescent (1.7%) did no longer complain about headache, 13 patients (22.8%) had mild, 24 (42.1%) moderate, 15 (26.3%) severe headache, and four (7.0%) reported the most severe headache. No association with headache diagnosis could be concluded (*χ*^2^ (12) = 12.152, *p* = 0.434), and the difference in intensity between genders was not significant (*χ*^2^ (4) = 8.183, *p* = 0.085).

**Fig. 4:**
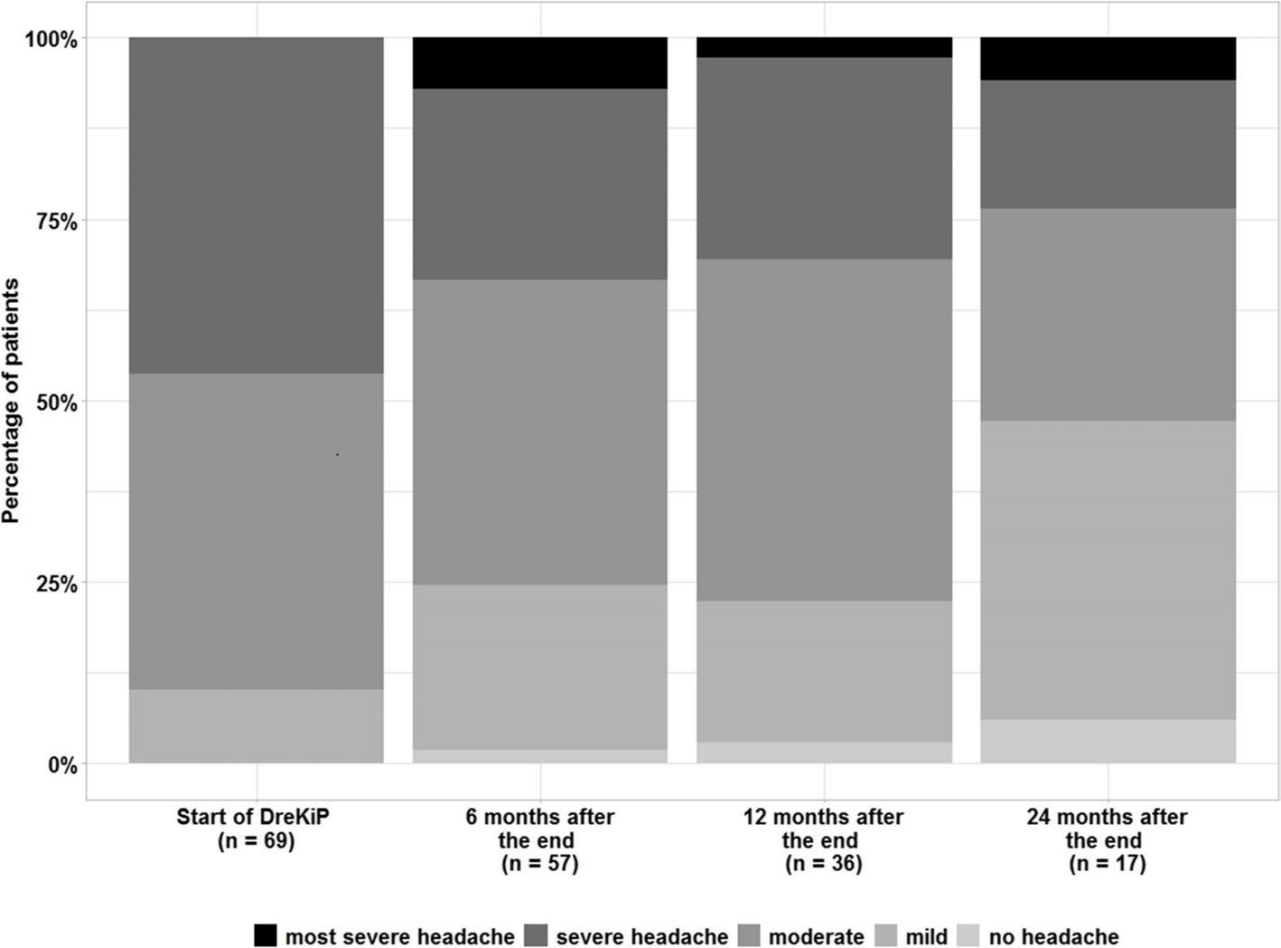
Headache intensity. (proportion of patients with headache intensity classified as “most severe”, “severe”, “moderate”, “mild” and “no headache” in the last 3 months) at baseline and follow up 6, 12 and 24 months after the program “DreKiP” (stands for Dresden children and adolescent headache program)

At T2, information on pain intensity was obtained from 36 (48%) patients. One (2.8%) had no headache, 7 (19.4%) mild, 17 (47.2%) moderate, 10 (27.8%) severe, and one (2.8%) the most severe headache (Fig. 4). No difference due to headache type (*χ*^2^ (12) = 17.135, *p* = 0.145) or gender (*χ*^2^ (4) = 7.722, *p* = 0.102) could be inferred.

Only 17 patients (22.7%) provided data on headache intensity at T3. Their data indicated no new trends as compared to T2.

The effects of time, headache diagnosis, and gender on headache intensity were explored using a linear mixed-effects model. As in the descriptive analysis, the effects of time and diagnosis were found to be significant (F = 4.089, *p* = 0.008, and F = 4.552, *p* = 0.006, respectively). A notable change in intensity was estimated after 12 months (about 30% decrease on average, *p* = 0.056). The estimated difference between baseline and 24 months was, on average, 40% (*p* = 0.025; Fig. 4).

### Analgesic medication at baseline and after group therapy

Initially, information about analgesic intake was provided by 72 patients (96%). Among them, 16 (22.2%) reported no analgesic medication intake for headache, 36 (50%) used analgesics more than once a month and 20 (27.8%) more than once a week.

At T1, medication-intake data were available for 65 (86.7%) participants, of whom 19 (29.2%) did not need analgesics, 36 (56.9%) used medication more than once a month, and 9 (13.8%) more than once a week.

**Fig. 5:**
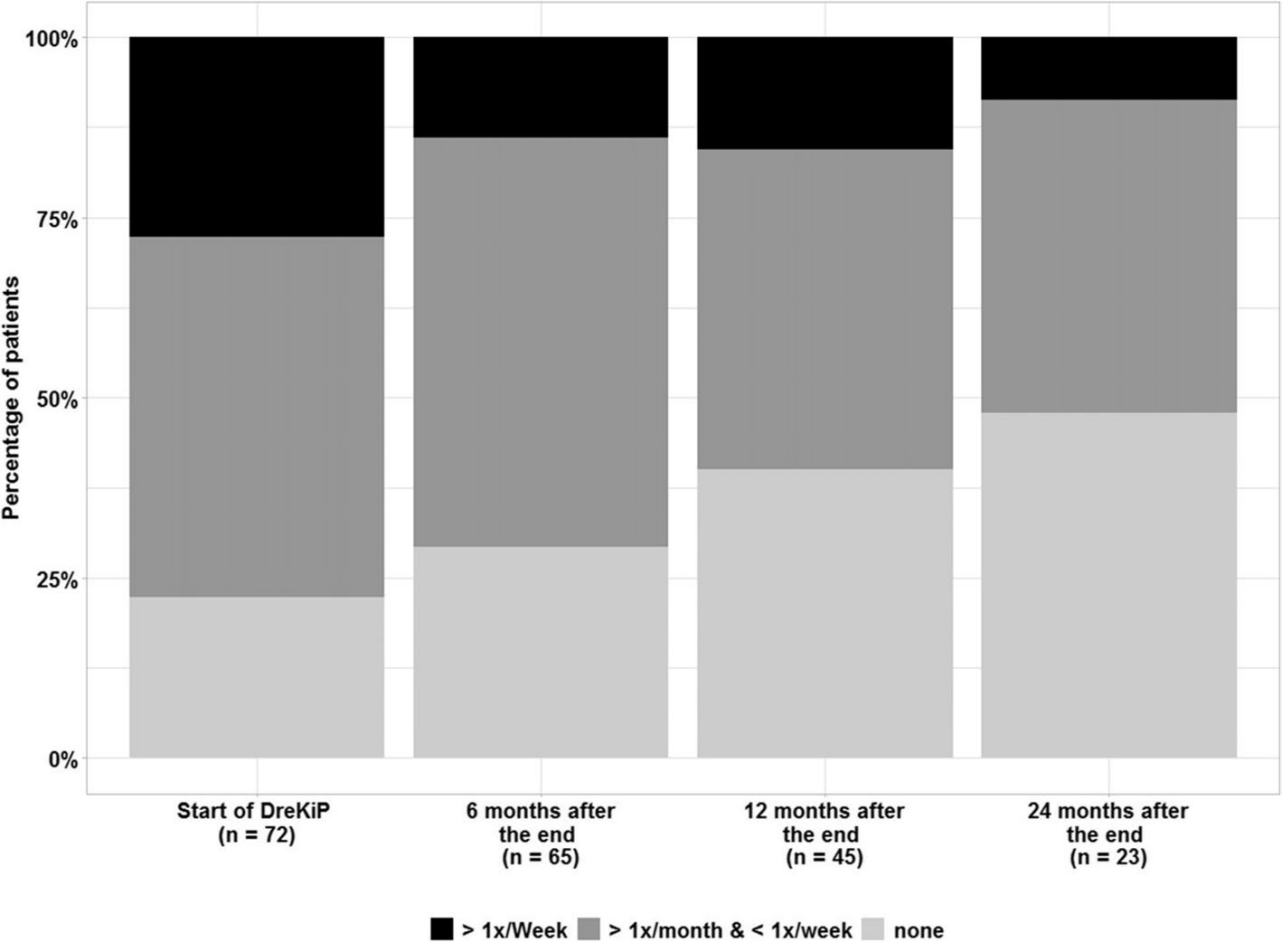
Acute Headache Medication intake at baseline and follow up, 6, 12 and 24 months after the program

At T2, 45 patients (60%) provided information on analgesics. Of them, 18 (40%) stated that they were not taking any medication, 20 (44.4%) reported taking it more than once a month, and 7 (15.6%) more than once a week (Fig. 5). Finally, at T3, data on analgesics were provided by 23 patients (30.7%). Two of them (8.7%) were using analgesics more than once a week, 11 patients (47.8%) reported that they were taking none (Fig. 5).

No statistically significant changes in analgesic intake, compared to the baseline, were found at either 6 months or 12 months time point (McNemar–Bowker symmetry test, *p* = 0.105, *p* = 0.128, respectively), however, the two-year difference to baseline was significant (*p* < 0.001). The association of analgesic intake with headache diagnosis and with gender at either time points was not significant.

### Headache related disability at baseline and after group therapy

At T0, PedMIDAS as measure of headache related disability of 75 children and adolescents were obtained. Scores ranged from 1 to 200, mean 40.51 (±39.25) and median 30, indicating outliers. Patients with chronic migraine presented higher than average scores, mean of 66.0 (±13.81). The second largest mean score of 40.0 (±7.97) was observed for the episodic migraine group. However, no overall association with headache diagnosis could be inferred (F(3,70) = 1.69, *p* = 0.177, log-scale).

At T1, PedMidas scores were obtained for 65 (86.7%) patients. The mean score 23.83 (± 24.93) was significantly lower than at the beginning of the program (V = 1465, *p* < 0.001). The maximum score of 120 and the median of 15 indicated a positive development as well. The difference in PedMidas due to headache diagnoses was not significant (F(3,61) = 1.167, *p* = 0.330, log-scale). As before, the chronic migraine group had the largest mean score of 36.6 (± 8.53), compared to other diagnosis groups.

**Fig. 6:**
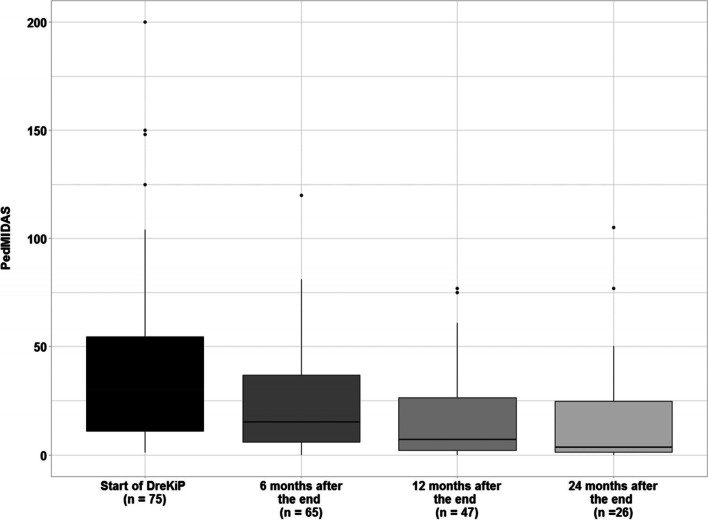
PedMidas. (Median) at baseline and follow up 6, 12 and 24 months after the program “DreKiP” (stands for Dresden children and adolescent headache program)

At T2 data revealed a further reduction in PedMidas scores. The scores of 47 patients (62.7%) who returned for the follow-up ranged from 0 to 77 (median = 7), and their mean 16.89 (± 20.35), was significantly lower than the baseline value (V = 947.5, *p* < 0.001; Fig. 6). As before, no overall association with headache diagnosis was found (F(3,43) = 1.987, *p* = 0.130, log-scale). The mean score of participants with chronic migraine was 41.6 (± 8.51).

At T3, PedMidas scores were obtained for 26 patients (34.7%). By then, the maximum score was 105, the mean was equal to 15.96 (± 26.16) and median = 3.5. Although these scores were not significantly different from the 12 months data (V = 89.5, *p* = 0.277), the improvement, compared to the start of the program, remained significant (V = 296, *p* < 0.001). As in the previous time points, no association with headache diagnosis could be inferred (F(3,22) = 1.196, *p* = 0.334, log-scale), with the mean score of participants with chronic migraine being the highest, 30.8 (± 10.04).

Further, the percentage change in PedMidas score over time was investigated using a linear mixed-effects model. According to the fit results, the scores decreased by 50% already in 6 months (*p* < 0.001), a decrease of about 70% was estimated to be achieved in 12 months (*p* < 0.001), and of almost 80% in 2 years (*p* < 0.001). Across all time points, the estimated scores for girls were on average about 90% higher than for boys (*p* = 0.024).

### Pain related disability at baseline and after group therapy

At T0, PPDI scores were available for 63 (84%) children and adolescents. The minimum and maximum scores were 18 and 57, respectively, with mean = 33.13 (± 8.82), and median = 34.

At T1, PPDI scores were obtained from 60 (80%) patients. This time, the scores ranged from 12 to 57, with the mean of 29.93 (± 10.85) and median of 30.

**Fig. 7:**
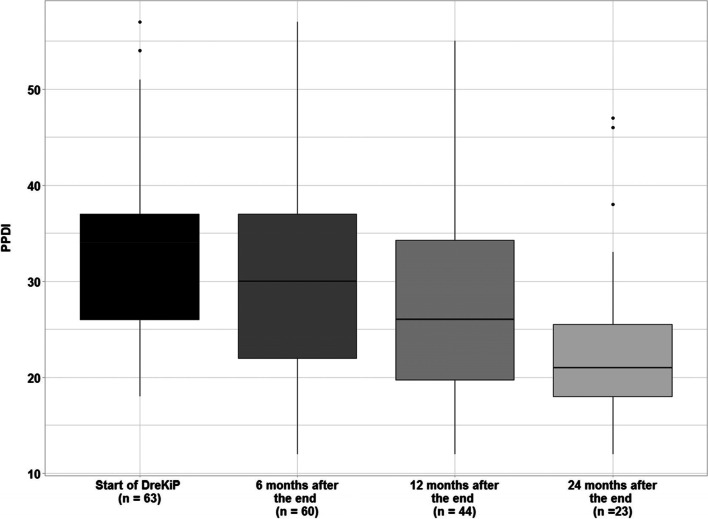
PPDI. (Median) at baseline and follow up 6, 12 and 24 months after the program “DreKiP” (stands for Dresden children and adolescent headache program)

At T2 scores were reported for 44 patients (58.7%). Although the score range stayed about the same, from 12 to 55, the new mean value of 27.91 (± 11.15) and median of 26 reflected a tendency of somewhat reduced pain disability (Fig. 7).

At T3 data were collected from 23 patients (30.7%). The scores, ranged from 12 to 47, with the mean of 23.61 (± 9.63) and median of 21 indicated a small reduction in pain disability (Fig. 7). At either time point, no significant differences in PPDI scores due to the headache diagnosis were found.

An additional analysis with linear mixed-effects models indicated an overall significant improvement in PPDI by about 25% starting from baseline to 24 months (*p* = 0.001), but implied a slightly different time dynamic in PPDI scores of boys and girls (*χ*^2^ (3) = 7.864, *p* = 0.049). Throughout the whole time, girls’ scores were estimated to be about 20% higher than those of boys (*p* = 0.022). By 6 months, boys’ scores decreased by 25% (*p* = 0.001), while girls’ scores stayed, on average, about the same (*p* = 0.3961). According to the model, a gradual decrease continued for both genders, leading to almost a 30% reduction in girls (*p* < 0.001) and 20% reduction in boys (*p* = 0.076) in 24 months after the program.

## Discussion

The results of the prospective study in an urban population in Germany indicate that the interdisciplinary multimodal group therapy program DreKiP is effective in improving headache-related limitation of daily functioning for children and adolescents with headache in a period of 6 to 12 and 24 months after the program.

61.3% of the patients were female, reflecting the more frequent occurrence of headaches in girls beginning with adolescence [7]. Most patients in the program were older than 14 years. This reflects the epidemiology of primary headache in childhood and adolescence, with prevalence increasing with age [6]. The majority of patients suffered from any type of migraine. Considering the psychosocial impairment that migraine has already in young patients, it seems compelling that these patients in particular have a need for intensified therapy [23].

At least one other disease was known for 60% of patients. Comorbidity of headaches and a range of diseases has been described in pediatric migraine [24]. It includes psychiatric disorders, head and neck injuries, cardiovascular disease, metabolic syndrome, asthma, sleep apnea and other pain syndromes and points towards multimorbidity beginning early in life. In adults all classes of comorbidities significantly increased the risk of progression from episodic to chronic migraine [25]. Children and adolescents with concomitant diseases also suffer more frequently from headaches [2]. Thus, it is obvious that a large proportion of patients treated in the program, show an increased risk for headache chronification.

In adult patients with migraine, interdisciplinary multimodal treatment programs have been shown to effectively reduce monthly headache days and improve daily function [26–28]. Unimodal or bimodal interdisciplinary treatment programs have been associated with improvement in headache frequency in children and adolescents [29–32]. *Treatment of patients with chronic migraine combining cognitive behavioural therapy with Amitriptyline was reported to be more effective than headache education combined with Amitriptyline* [33]. This underlines the impact of cognitive behavioral therapy in pediatric migraine therapy but points already to the advantages of using different therapy modalities. However, there is limited data on the longterm treatment effects of interdisciplinary treatment programs for children and adolescents with headaches [23]. Especially, there are only a few prospective studies published, mainly investigating all types of pain in children and adolescents [34–36]. A study investigated 50 children with chronic headache aged 10-19 years attending an interdisciplinary therapy program (8 h/day, 5 times/week, 2-7 weeks) [35]. At 6-8 week follow-up Headache Impact Test-6 reduced significantly, disability and school participation improved. Another inpatient interdisciplinary treatment program for pain has been investigated using a waiting control group, each 52 patients [34]. Significantly more children in the program compared to controls improved in pain intensity, disability, school absence directly after the program. However, a unique feature of our treatment program is a follow up “booster” treatment 6 and 12 months after the training. The content of the therapy program consists also of effects on lifestyle and personal attitudes. More mindfulness for the balance of effort and relaxation as well as regular physical activation are the focus. This long-term behavioral adjustment is more likely to succeed through repeated training, even at longer intervals. A comparable concept has not yet been described for children and adolescents with headaches. Kroner et al. used combined cognitive behavioural therapy and Amitriptyline with a protocol of booster therapies twice monthly for at total of 20 weeks [33]. Nevertheless, the potential additional effect of booster sessions compared to the program without booster therapy has not been evaluated. In adults, booster sessions on self-management interventions for chronic musculoskeletal pain have been studied with randomised controlled trials of low quality only [37] reporting little evidence of effectivity in musculoskeletal pain relief. However, no data are available for children and adolescents with headache on this matter. In pediatric migraineurs age dependent differences have been found in brain regions relevant for sensory and affective functions [38]. This potential of neural plasticity in young migraineurs could have implications for the success of multimodal therapies. Therefore, further prospective studies are needed.

Compared to the sparse data available from other therapy programs, our patient population shows significant improvements in daily function 6 and 12 months and a subgroup of patients even 24 months after the end of the program. This is accompanied by a significant reduction in the number of headache days 6 and 12 and 24 months after the end of the program. This is also reflected in the reduced pain disability after the program. However, it should be noted that the PPDI is valid as a measure of chronic pain impairment in children [39] but does not capture the extent of impairment as well as the PedMidas, especially for episodic headache patients. However, since 24% of the children also reported concomitant pain other than headache, the PPDI can also describe the extent of the overall development. For the chronification of migraine, a connection with comorbid pain has been described in adults [40]. The program described here, can have additional effects due to the versatile therapy content. Further studies are required to make a precise statement on this.

To our knowledge, this is the first prospective evaluation of an interdisciplinary multimodal outpatient program for children and adolescents with recurrent or chronic primary headaches. Headache related disability, headache frequency and overall pain disability were significantly reduced 6 and 12 and 24 months after the program. DreKiP showed to be effective in improving headache frequency and headache related disability.

However, our study has several limitations. First of all, there is a number of patients with incomplete or missing data. We investigated whether patients without follow up showed different baseline data for headache frequency and disability (Supplementary Fig. 1, Supplementary Table 3). Baseline data were not significantly different between the groups. Therefore, we assume other factors as critical. Interestingly, we observe an association between age ≤ 14 years and follow up but not with school type or gender. Another complicating point is that we studied a mixed group of headache patients. Since chronic tension-type headache and episodic migraine might have different pathophysiologies, different therapy focuses might be necessary. At this point, no conclusions can be drawn, whether the therapy content is more suitable for a particular diagnosis group. Another limiting factor is the missing control group. Future evaluations should include a control group of children and adolescents in outpatient pediatric care.

An important fact is, that we see gender-specific differences in the headache related disability and also in the reduction of it following the group therapy. This point has not been taken into account yet and should be investigated in the future.

## Conclusions

Our study demonstrates that the interdisciplinary multimodal group therapy DreKiP is a valuable and sustainable treatment for children and adolescents with frequent headaches. Further establishment and investigation of the effectiveness of multimodal therapies should be encouraged. Not least because relevant social and economic disease burdens arise already in childhood and adolescence due to frequent headaches.

## Supplementary Information

**Additional file 1: Supplementary Fig. 1.** Baseline data for headache disability (PedMidas) of patients who provided follow up data compared to baseline data of patients who did not provide follow up data. **Supplementary Table 1.** Age distribution of patients in the program (yrs…years). **Supplementary Table 2.** School type of patients in the program. **Supplementary Table 3.** Mean values of the groups (with/without follow up data) for headache disability (PedMidas) and headache days are not significantly different at the beginning of the program. **Supplementary Material 1.** Therapy modules of the interdisciplinary multimodal treatment program for children and adolescents with headache (DreKiP).

## Data Availability

The datasets used and/or analyzed during the current study are available from the corresponding author on reasonable request.

## Abbreviations

MIDAS: Migraine disability assessment score
fMRI: Functional Magnetic Resonance Imaging
HIT-6: Headache Impact Test
SPSS: Statistical Package for the Social Sciences
BMI: Body Mass Index
cM: Chronic Migraine
eM: Episodic Migraine
