# Supplementary material for: Functional improvement in children and adolescents with primary headache after an interdisciplinary multimodal therapy program: the DreKiP study
Source: J Headache Pain. 2022 Aug 25;23(1):109. doi: 10.1186/s10194-022-01481-1 (PMC9404663; doi:10.1186/s10194-022-01481-1)
Supplement: Supplementary file 1 — Additional file 1: Supplementary Fig. 1. Baseline data for headache disability (PedMidas) of patients who provided follow up data compared to baseline data of patients who did not provide follow up data. Supplementary Table 1. Age distribution of patients in the program (yrs…years). Supplementary Table 2. School type of patients in the program. Supplementary Table 3. Mean values of the groups (with/without follow up data) for headache disability (PedMidas) and headache days are not significantly different at the beginning of the program. Supplementary Material 1. Therapy modules of the interdisciplinary multimodal treatment program for children and adolescents with headache (DreKiP). [file 10194_2022_1481_MOESM1_ESM.docx]

Supplementary Tables

**Supplementary Table 1.**

|  | | **%** | **N** |
| --- | --- | --- | --- |
|  | 9-10 years | 6.7 | 5 |
|  | 11-13 years | 21.3 | 16 |
|  | 14-16 years | 57.3 | 43 |
|  | 17-19 years | 14.7 | 11 |

**Supplementary Table 2.**

| N=68 | | **%** | **N** |
| --- | --- | --- | --- |
|  | primary school | 11.8 | 8 |
|  | 6-year-secondary schools (secondary school level I certificates) | 32.4 | 22 |
|  | 8-year-secondary schools (university entrance level II certificates) | 42.6 | 29 |
|  | vocational secondary school | 7.4 | 5 |
|  | private or special schools | 5.9 | 4 |

**Supplementary Table 3.**

Mean values of the groups (with/without follow up data) for headache disability (PedMidas) and headache days are not significantly different at the beginning of the program (t-test for independent samples, equality of variance proven with Levene's test).

- PedMIDAS: p = 0.416

|  | + follow up data | - follow up data |
| --- | --- | --- |
| N | 75 | 36 |
| Gender female/male | 50 (66.7%) / 25 (33.3%) | 25 (69.4%) / 11 (30.6%) |
| Age mean | 14.20 | 15.42 |
| PedMIDAS mean (SD) | 40.51 (39.25) | 47.52 (39.32) |
| Headache days mean (SD) | 43.74 (33.44) | 47.18 (31.10) |
|  |  |  |

- Headache days: p = 0.618

Supplementary Material 1

Therapy modules of the interdisciplinary treatment program for children and adolescents with headache (DreKiP)

The DreKiP therapy program is offered four times a year and is conducted over 2 to 3 months each time. Specifically, the first cycle lasts from January to March, the second from April to June, the third from late August/early September to October, and the fourth from October to December. Only patients who fulfill the inclusion criteria of the program are admitted to it. Inclusion criteria include an age between 9 and 18 years old; a confirmed diagnosis of a primary headache disorder according to the ICHD‐III criteria; the persistence of the symptoms for at least 6 months; headache-related limitations in school attendance, daily activities, quality of life and motivation. Headache frequency, headache-related disability, and other patient related outcome measures are assessed at the beginning of the program and 6, 12, 24 months after its completion. This means that follow up data are collected in March, June, October and December, respectively. Thus, we do not collect these data in or directly after the usual summer holiday period, which in the local state (Saxony) occurs between July and August. Rather, 2 of the 4 evaluation time points occur in the seasonally more headache-prone period from fall to winter.

Module 1 - Education

At the beginning of the program the attending physicians, the patients and parents are introduced. the therapists involved in the program are named with a foto and specialty. The patients introduce themselves with name, age, city of origin, class, school, how are things today, favorite animal and hobbies. After that follows a short introduction of the parents with personal motivation. Following this, patients and parents are separated. The pediatrician works with the patients and presents the group rules. Then patients are guided to work out with their own type of headache, treatment options for non-drug an medication therapy. In parallel, the parents are instructed by a doctor, experienced in headache therapy, on various forms of headache and the headache classification. The available therapies are discussed. Expectations as well as disease conceptions which the parents have developed, are put in the context of the current pain medicine. The possibility to exchange between the parents among each other during each module is supported by the physician.

Module 2 – Stress management

A pediatric psychotherapist works out headache-related stress factors with the patients. Using the biopsychosocial pain model, stressors are classified and coping mechanisms are sought. Existing strategies are reviewed and new ways of coping with stress, such as relaxation/defocusing techniques are introduced and trained. In parallel, the parents work out with a doctor on how to deal with their children's headache. On the one hand lifestyle factors such as irregular daily routines, too little rest periods, etc., which can increase the incidence of headache pain, are reflected and individual possibilities for change are discussed. On the other hand, the management of specific headache situations is trained.

Module 3 – Relaxation techniques

As a continuation of the previous module, a relaxation therapist reflects with the patients applied strategies for relaxation in stressful situations in everyday life. Depending on the age of the patients, various relaxation techniques such as au- togenic training and progressive muscle relaxation according to Jacobson will be theoretically introduced and practiced. In addition, techniques such as biofeedback and neurofeedback are introduced and experienced. These possibilities of regulation and self-efficacy training will be developed as a resource. Time for learning and practicing together is provided. In parallel, parents are trained in the various techniques and are invited to apply them in practice as well.

Module 4 – Physical activation

Physiotherapy focuses on the physical activation of the patients. The aim is to (re)awaken the fun of movement. Endurance sports as a headache prophylaxis is presented, and training possibilities are tested. The connection between posture, muscular tension and tension headache is explained. For children up to 12 years of age the first part is held together with the parents. For all patients, exercises for muscle relaxation and strengthening are introduced and implemented with the aim to restore adequate muscle tone, especially in the shoulder-neck area. Children 13 years and older learn the exercises without the presence of their parents.

Module 5 – Climbing and self efficacy

Climbing therapy is another form of physical activation. Climbing strengthens the entire musculature, develops coordinative skills, but also social skills such as trust and integration in a group. These positive effects on physical and mental health are already being discussed in the various mental illnesses, but also for hemophilia patients. In addition, the personal beliefs of the patients and thought patterns are promoted. The self-efficacy becomes highly improved. The therapy is based on the concept: "Out of the comfort zone,into the learning zone, avoid the panic zone". After each therapy, patients reflect with therapeuts the training and assess their status within the zone model during the climbing activity. This helps further reflections on behaviour and personality.

Module 6 – Defocussing and resources

Art therapy works with different materials that can be experienced by the senses, primarily with the aim of defocussing. The task serves the stimulation of a result-open creative process, which includes expression, change of perspective and the recognition of unknown resources. The parents meet with a physician and process the new knowledge, reﬂect on the connection between their children's headaches and life situation.

Module 7 – Relaxation techniques – repetition and training

Module 8 – Smell therapy and repetition stress management

Patients and parents get an introduction on effects of odors on headache. The basic physiology of perception of smells is presented and experienced in short experiments. Based on neuroanatomic connections between olfactory system and brain regions, which functionally belong to the "pain network“, for example, the amygdala, odors might influence pain. Structured training with scents has been shown to influence pain by increasing the mechanical pain threshold. Patients get the possibility to use pleasant odors (sniffin sticks) as coanalgesic.

Booster module - 6 and 12 months after the therapy program

Booster modules are performed as group meetings (patients and parents). Here the current individual conditions of the patients are reported and solution strategies for problems in implementing the therapy content are developed. In detail, contents of stress coping strategies are repeated in the group. In the discussion, individual stressors and existing coping strategies are reflected upon. Further possibilities of favorable stress management are implemented. In addition, the extent of regular physical activation of the patients is examined. The aim here is to develop awareness of the need for physical activity in everyday life. Concrete sports opportunities are determined with the patients. The everyday transfer of the learned relaxation techniques is reported. Difficulties in implementation are reflected and supportive measures such as videos and daily plans with relaxation units are planned with the patients. 12 months after the end of therapy, another booster therapy session of the group takes place and again the essential therapy contents are checked for everyday life transfer. At all visits, the current presentation of headaches is recorded, the frequency and analgesic intake. If necessary, medication corrections are made, especially acute medication and, in individual cases, prophylactic headache medication.

*Some of the contents may sound challenging, i.e,. the climbing therapy. Having educated climbing therapists running these activities, allows them to run smoothly, with most of our young patients having a very positive attitude toward them. Indeed, even those who fear the height can be engaged and show their usefulness by securing from the ground those who are climbing on the wall. Hence, each participant plays an active role and has self-efficacy. In fact, the costs of a climbing therapy exceed those of a psychotherapist by <20%, while evoking strong perceptions and a long-lasting memory in the patients. Standardization of climbing therapy is possible as much as standardization of physio- or psychotherapy. Nonetheless, we agree that it would be very valuable to compare single treatment approaches directly.*
